# Health provider and service-user experiences of sensory modulation rooms in an acute inpatient psychiatry setting

**DOI:** 10.1371/journal.pone.0225238

**Published:** 2019-11-21

**Authors:** Skye P. Barbic, Nicole Chan, Amanpreet Rangi, James Bradley, Rachal Pattison, Kerri Brockmeyer, Sandy Leznoff, Yojo Smolski, Gagan Toor, Blaine Bray, Adelena Leon, Malcolm Jenkins, Steve Mathias

**Affiliations:** 1 Faculty of Medicine, University of British Columbia, Vancouver, British Columbia, Canada; 2 Department of Occupational Science and Occupational Therapy, UBC, Vancouver, British Columbia, Canada; 3 Providence Health Care, Vancouver, British Columbia, Canada; 4 Department of Psychiatry, UBC, Vancouver, British Columbia, Canada; 5 Centre for Health Evaluation and Outcome Sciences, Vancouver, British Columbia, Canada; 6 Foundry, Vancouver, British Columbia, Canada; 7 Department of Psychiatry, St. Paul’s Hospital, Vancouver, British Columbia, Canada; University of California Los Angeles, UNITED STATES

## Abstract

**Background:**

Sensory modulation rooms (SMRs) are therapeutic spaces that use sensory modulation concepts and strategies to assist service users to self-regulate and modulate arousal levels. SMRs are increasingly being explored as strength-based and person-centered adjuncts to care for people receiving inpatient psychiatry services. The aim of this study is to understand health provider and inpatient service user perceptions on the use of SMRs on acute psychiatric units.

**Methods:**

We conducted semi-structured interviews with ten service users and nine health providers (four occupational therapists and five nurses) regarding their experiences of the SMRs located on three acute inpatient units in a large urban tertiary care hospital. We audio recorded and transcribed the focus groups and used thematic analysis to analyze the data.

**Results:**

Our results suggested four common themes amongst health provider and service user experiences of sensory modulation rooms: (1) service user empowerment through self-management, (2) emotional regulation, (3) an alternative to current practices, and (4) health provider and service user education.

**Conclusion:**

Our study supports the ecological utility of SMRs as person-centred adjunct therapeutic space viewed positively by both service users and health providers. This understanding of SMRs is critical for future service design, research and policy aimed at improving the service user experience and care for this population. Future research is needed to validate the experience of the SMRs with other patient groups and health providers.

## Introduction

The use of restraints and seclusion within mental health settings has long been considered controversial. Throughout the mid-19^th^ century, asylums within the United States became increasingly overcrowded. As a result, methods of behavioural control for asylum residents became a primary concern [1, 2]. Mechanical methods to control problematic behaviours were perceived to be necessary and consequently a variety of restraining devices were developed [1–3]. The alternative to physical restraint was seclusion, which was generally considered the more humane approach at the time [1]. By current societal standards, seclusion and restraints are less than ideal methods of behavioural control. Limited research exists to support such tactics, with the exception of relatively extreme and rare situations where individuals are at risk for hurting themselves or others [4, 5]. Seclusion and restraints have been associated with traumatic long-term mental health and social impacts for a person [1, 6–9]. Consequently, finding alternatives to restraints and seclusion is a priority for psychiatry and mental health. One new option for inpatient settings is the use of sensory modulation rooms (SMRs) [3, 10–14].

Sensory modulation is the way individuals regulate and organize their response to sensory input received from their environment [11, 15, 16]. Recently, sensory modulation has been defined by Brown and colleagues (2018) as ‘a twofold process’ [16] that “originates in the central nervous system as the neurological ability to regulate and process sensory stimuli; this subsequently offers the individual an opportunity to respond behaviorally to the stimulus” (p.9). Furthermore, sensory modulation is described as something that individuals are constantly doing throughout the day, whether they are aware of it or not [17]. As individuals become more aware of how they are personally affected by certain environmental stimuli (for example, specific sounds or smells), they can develop strategies to regulate the nature of their responses to sensory input levels [3, 18, 19]. These strategies can enable the individual to achieve and maintain optimal function in everyday life, as well as to adapt to the challenges faced in daily life [3, 11, 17, 20]. As such, sensory modulation has a strong basis for therapeutic use across all mental health settings [10, 11, 13, 20–22].

SMRs are broadly defined as therapeutic spaces specifically designed and used to support sensory-based therapeutic interventions [13, 23]. Sometimes referred to as “sensory rooms” or “comfort rooms”, [24, 25] SMRs were originally constructed in the 1960s to provide therapeutic sensory stimulation for individuals living with severe disabilities and learning difficulties. However, their application has since been expanded to dementia care [3], school settings [26], and most recently, psychiatric care [10, 11, 20]. Within the context of inpatient psychiatry, SMRs have been used in the United States, New Zealand and Australia to promote self-soothing strategies in times of distress [11, 20, 23, 27, 28]. Such approaches include the use of sensorimotor activities and sensory modalities, environmental modifications, and support in developing personalized self-regulation strategies to cope with daily stressors [12]. Furthermore, such interventions have been shown to be beneficial for psychiatric inpatients [3, 27]. For example, one literature review found that 85% of mental health service users reported a reduction in distress when engaging in sensory interventions in SMRs [3]. Moreover, these interventions were recognized as non-invasive, self-directed, and empowering strategies that may reinforce recovery-oriented and trauma-informed mental health practices [3].

The use of SMRs has also been found to generally reduce the incidences of seclusion, physical restraints and pharmacological interventions for this population [1, 24]. In particular, SMRs have been shown to decrease a service users’ self-perceived arousal level by promoting multi-faceted and self-soothing strategies, thereby reducing the need for external precautions [9, 11]. Moreover, SMRs have also been shown to strengthen the therapeutic relationship and promote collaboration between the healthcare provider and the service user [10, 18, 28]. However, in all of the studies mentioned above, limited qualitative information is provided to describe the experiences of service users and the providers using SMRs.

Our team, which included inpatient mental health experts, identified that more information was needed about the service user and staff experience regarding SMRs for inpatient psychiatry settings. Our context of study is an urban setting in Vancouver, Canada that is known for high rates of inpatient utilization related to mental health and substance dependence [29, 30]. We are unaware of any published literature understanding the experiences of SMRs for this context of use (inpatient setting within an urban tertiary care hospital). This is particularly problematic due to the healthcare and opioid crisis currently impacting this region, with emergency room and inpatient visits by people experiencing a mental health crisis and/or an illicit drug overdose rising each year [31, 32]. More specifically, the centre of study, St. Paul’s Hospital, receives approximately 12,500 mental health and substance-related emergency department admissions on an annual basis [33]. In efforts to explore viable interventions and treatments for mental health and addictions, and improve the overall service user experience, St Paul’s Hospital has introduced SMRs on three acute mental health units and one neuropsychiatry unit. The aim of this study is to explore the experiences of health providers and service users using SMRs in this context of use.

## Methods

### Design

This study was approved by the Providence Health Care Behavioral Ethics Board (H17-01136). The Consolidated Criteria for Reporting Qualitative Research (COREQ) was used to guide the study design and reporting. We used a qualitative descriptive study design, guided by phenomenology, using 1:1 interviews with staff and service users to evaluate the acceptability, implementation, and experiences of the SMRs. The study’s Principal Investigator (SB) and two graduate students (AR and NC) completed all consenting procedures and interviews. The research team did not know the participants before the study start. We communicated the interviewer characteristics (credentials, place of work/study, qualifications to conduct the research, ethics certificate number, reasons and interests in the research topic) to hospital leadership and key stakeholders at the recruitment site prior to the study start.

### Theoretical framework

The methodological orientation to the study was qualitative thematic analysis. This framework was chosen over content analysis because the frequency of occurrences was not the main goal of the analysis. Rather, we were interested in the perspectives of health providers and service users and understanding how the sensory modulation rooms impact care [34].

### Participant selection

We sampled participants from three inpatient mental health units located in an urban academic tertiary hospital located in Vancouver, Canada. We sampled service-users receiving care from the inpatient unit and health providers working on these units. For the purposes of this study, we did not recruit service-users or health providers from the inpatient neuropsychiatry unit, as the care provided by this unit was deemed by clinical leaders as unique and suitable for a separate study.

### Method of approach and sample size

For health providers, we used purposive sampling to recruit three to five staff from each unit. We provided study information sheets to all health providers working on the wards, including the contact information of the research team. We asked interested participants to call or email the study team to learn more about participation. Participants were: adults aged 19 years or older, those willing to provide written consent, providers of inpatient mental health services at the recruitment centre and had prescribed use of the modulation room as an intervention at least one time.

For service-users, we asked the clinician nurse lead on the unit to share information about the study with three to five service users on each ward who had used the SMR, were well enough to learn more about the study, and able to speak to a research team for 30–60 minutes about their experience with the SMR. We asked the clinician nurse lead to provide potential participants with a study information sheet and the contact number to reach the study team to learn more about the research. Participants were: adults aged 19 years or older, willing and able to read and respond in English, willing and able to provide informed consent, receiving inpatient mental health services from the acute care hospital at the time of study and were using sensory modulation during their inpatient stay. Participants that were excluded from the study were those identified by health providers on the ward to be unable to safely complete the interview or consent.

### Setting

As noted above, the study was conducted on three inpatient mental health units. All units have capacity for 17 to 20 beds and care is provided by interdisciplinary teams that include psychiatry, nursing, occupational therapy, addiction therapy, pastoral services, dietitian, pharmacy, and social work disciplines. Each unit’s SMR ranges from 75–125 square feet and consists of sensory tools that enable clients to modulate their arousal levels by facilitating either calming or energizing effects. These tools include visual, auditory, olfactory, vestibular and tactile devices such as constellation projectors, bubble light tubes, projected images, and printed light and window screens. Other examples of visual tools available in the room include various light-up modalities such as lava lamp devices or alternative lighting systems. Auditory tools primarily consisted of radio or iPod setups, and white-noise/wave sound machines. Different essential oils and other scented modalities were available for use in a locked cabinet in order to engage the olfactory sense. Alternative seating methods (via mats and beanbag chairs) in addition to rocking chairs were available in both rooms. Lastly, various fidget tools were available throughout the rooms and within the locked cabinet as a method to engage the tactile sense. On average, service users spend 15–60 minutes in the room at one time.

### Procedures

After reviewing study details with participants, we obtained written informed consent, approved by Providence Care Ethics Board (#H17-01136) from all participants. After contact by the potential participant, the research team offered to meet in person to provide a copy of the consent documents and review study details. We asked participants to review the documents, ask any questions they may have, and sign the consent form immediately prior to the interview. During this process, the Principal Investigator (SB) was responsible for judging the potential participant’s capacity to consent on his/her/their own behalf. Of note, the Principal Investigator (SB) explaining the study and obtaining consent was not involved in providing direct care to the participants.

#### Informed consent for staff and service users

The first pages of the research package contained two copies of the informed consent form (ICF), including the study description, objectives, procedures, risks and benefits, and the contact information for the researchers for any questions or concerns. We provided participants with a quiet room on the unit to review the consent and gave them as much time as needed to ask any questions and make an informed decision to participate. We informed all participants that their decision to participate or not would have no bearing on the current or future clinical and health services they would receive at the institution of care.

#### Presence of non-participants

Only study participants and members of the research team (SB, AR, NC) were present at the time of the interviews. Once consent was obtained, we conducted interviews with participants (health providers and service users) and asked them about their experiences with the SMR. The research team used a semi-structured interview (see Table 1) for all the interviews and we collected field notes during and after the interviews. The interview schedule was developed after a review of the literature and input from the study team that included patient partners, health providers, and hospital decision-makers. We compensated participants for their time with a modest honorarium based on an estimated 60 minutes to complete the interview. Transcriptions of audio files and field notes were completed by study team members present at interviews (NC, AR, SB). Aggregate data for preliminary and final analyses were available to the entire study team for review and interpretation.

**Table 1:** Interview questions for study participants.

Service Users (n = 10)	Health Providers (n = 9)
1. How did you first hear about the sensory modulation room?	1. How did you first hear about the sensory modulation room?
2. How often have you used the sensory modulation room?	2. How often have you recommended the sensory modulation room to patients?
3. Why did you decide to try it?	3. Why did you decide to try recommending it?
4. Can you please describe your mood before entering the sensory modulation room?	4. Can you please describe your patients’ mood before using the sensory modulation room?
5. Can you describe your mood after using the room?	5. Can you please describe you patients’ mood after using the room? a. Did you notice any changes? If so what were they? b. If not, why do you think that is?
6. Which aspects of the room did you use most? a. How did you use them? b. Why?	6. Which parts of the room did patients report using most? a. Why do you think this is?
7. What parts of the room did you use the least? Why?	7. What parts of the room did patients report use the least? a. Why do you think this is
8. How long would you typically spend in the sensory modulation room?	8. How long would your patients typically spend in the sensory modulation room?
9. Were there any parts of the room that you would change?	9. Were there any parts of the room that you, as a clinician, would change?
10. Has the room helped you during your stay at St. Paul’s? a. Why or why not? b. In what ways?	10. Has the room helped your patients during their stay at St. Paul’s a. Why or why not? b. In what ways?
11. How do you feel the staff view the room? Useful? Not useful? And why?	11. How do you feel the staff view the room? Useful? Not useful? And why?
12. Do you feel like all patients could access the room if they wanted/needed to? a. Why or why not? b. What changes do you think would be needed?	12. Do you feel like all patients could access the room if they wanted/needed to? a. Why or why not? b. What changes do you think would be needed?
13. What was your initial impression of the room? a. Has that impression changed at all?	13. What was your initial impression of the room? a. Has that impression changed at all?
14. Would you recommend the room to other patients? a. If Yes, please describe why? b. If No, please describe why?	14. Would you recommend the room to future patients? a. If Yes, please describe why? b. If No, please describe why?

#### Data analysis

We used a data-driven inductive approach to generate themes from the data to allow for flexibility in exploring this topic and adequately representing health provider and service user perspectives [35]. The thematic analysis was guided by the study research question. Two members of the research team (NC, AR) organized and manually coded the data according to semantic themes. Transcriptions were organized and separated into “Health Provider” and “Service User” files. The researchers then analyzed all of the health provider interviews to reveal four main semantic themes. An identical process was completed for the service user interviews. We identified main themes from health provider and service user interviews. We then cross-referenced these content themes for triangulation and to ensure the themes captured the experiences of participants and the impact of the sensory modulation room on quality of care and recovery. The results were then presented to a subset of health providers and service users for validation (n = 8).

## Results

A total of ten service users and nine health providers participated in this study. At the time of the interview, all service user participants were admitted to one of three adult inpatient psychiatric units. Service user participants were between 23 and 73 years of age (mean 43, SD = 15), consisted of seven males and three females, and had a range of primary diagnoses such as anxiety, depression, bipolar, schizophrenia, and schizoaffective disorder. The health provider participants were five nurses and four occupational therapists who currently worked in the acute care hospital and had previous experience using the SMRs with acute inpatient service users. The average age of service providers was 41 (SD = 11), with four providers identifying as male and five as female.

**Fig 1:**
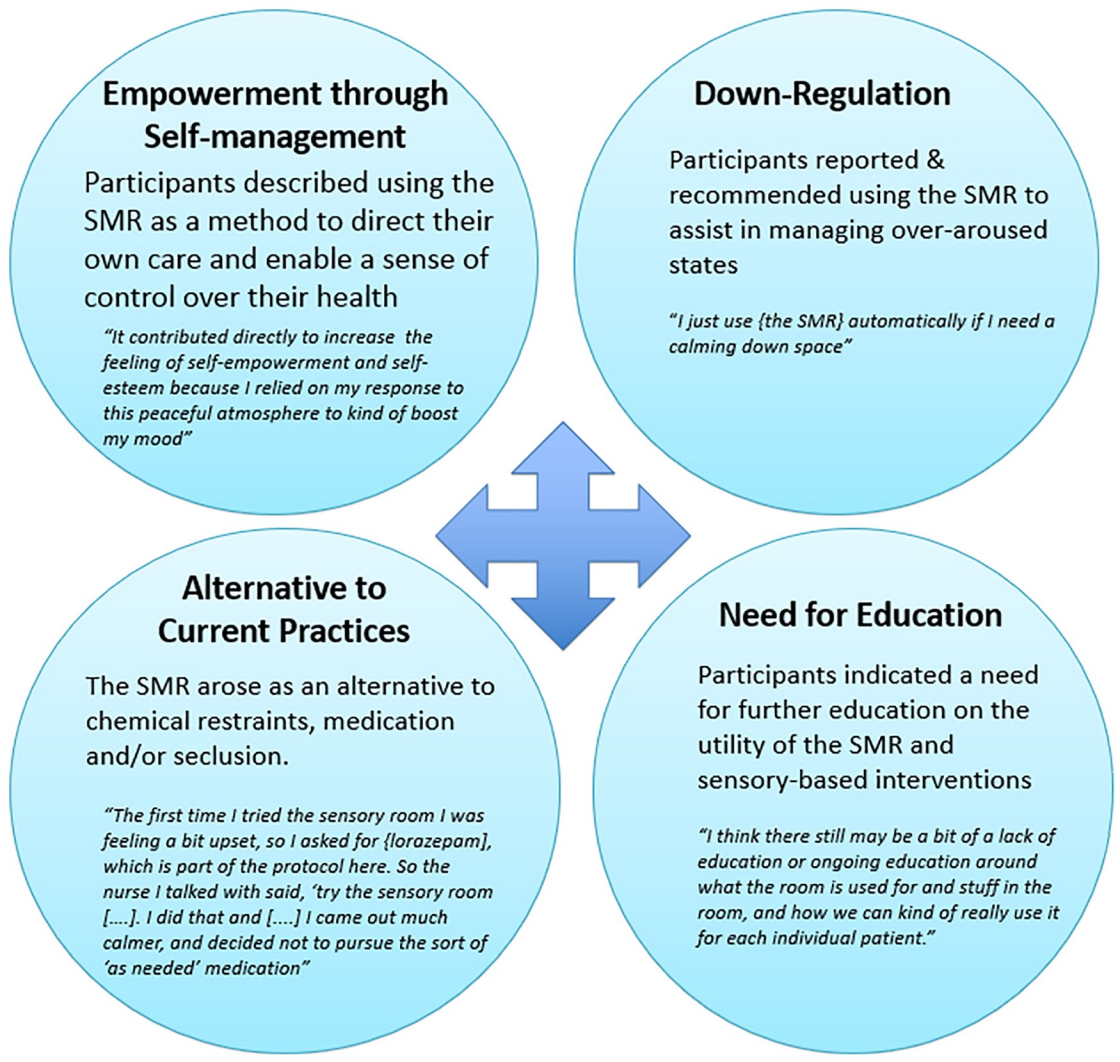
Themes and sample quotes extracted from the qualitative analysis of health providers and service users reporting their experiences with sensory modulation rooms on inpatient psychiatry settings.

As shown in Fig 1, four common themes emerged: 1) service-user empowerment through self-management, 2) emotional regulation, 3) an alternative to current practices, and 4) health provider and service user education.

### (1) Service user empowerment through self-management

The most prevalent theme amongst both service user and health provider interviews was utilizing the SMR to empower patients and enable self-management strategies to enhance their care experience. Service users reported using the SMR as a method to self-direct their own care and allowed for more control over their health. Service users described the SMR as a safe personal space, in a traditionally challenging environment, that allowed for an increased sense of empowerment and self-efficacy. Despite being on an inpatient unit with certain rights and privileges revoked under the Mental Health Act 1996 (BC), one service user stated:

*P3: “It contributed directly to increase the feeling of self-empowerment and self-esteem because I relied on my response to this peaceful atmosphere to kind of boost my mood–so I wasn’t relying so much on the medication to boost my mood–so it kind of increased the feeling of self-efficacy*.”

Multiple health provider participants corroborated this sentiment. One nurse stated:

*C1: “When [the patients] first come in, [the nursing staff] take away their clothes; take away their privilege to go out freely. You know, they’re certified—it’s very controlled*.”

Health providers acknowledged that the inpatient setting was a difficult environment to provide the safest care to the service users while also promoting wellness. The challenging parameters (e.g., locked setting, acutely ill service users, high staff turn over) associated with this inpatient setting were acknowledged by most health provider participants and the SMR was identified as a way to give dignity and control back to the people receiving services on the unit.

*CI: “I feel like it’s definitely a difficult situation in the sense of how to provide safest care to the patient without re-traumatizing them and forcing a sense of control. So I think that this sensory modulation room [gives] patients a sense of self-control back to them, because it is a space that is actually designed specifically for wellness and mindfulness. And it’s very tangible. You know, you can tell someone to go try deep breathing or whatever in their room, but then…to tell them to go to a sensory modulation room—it’s a very tangible, concrete kind of space for [the patients] to just, kind of, practice different coping strategies and try to decrease their distress*.”

A second health provider (nurse) echoed this sentiment and emphasized that SMRs can teach personal resilience in challenging circumstances:

*C2: “I think [the room is] a more sustainable way to match a lot of the issues the people are having, in terms of just building resilience and kind of empowering people to kind of improve their own circumstance and…I think it teaches a kind of personal resilience in terms of dealing with what they’re actually feeling and not necessarily just using chemical means of doing so*.”

Both groups of participants highlighted how acute inpatient restrictions challenged how recovery-, health-, or wellness-oriented care could be. SMRs were identified as a welcome space for exploring or re-discovering hope, empowerment, and self-agency.

### (2) Emotional regulation

A second theme that emerged across both service user and health provider interviews was utilizing the room as a method for emotional regulation, specifically down-regulation. Most (nine out of the ten service user participants interviewed) reported feeling “anxious” or increasingly “stressed out” prior to using the room, and described using the room to “relax”, “feel calmer”, or “chill out”. One service user participant reported integrating the room into their daily routine as a proactive method of dealing with anxiety while admitted as an inpatient:

*P3: “I just now use it automatically if I just need a calming down space*…”

This participant also reported that they have recommended it to others on the unit as a way to deal with anxiety or stress:

*P3: “Well sometimes people just say: ‘Oh, I’m feeling upset or worried about this and that’ and I [say]: ‘Well try out the [sensory] room. I’ve started using it, and it helps me when I’m starting to worry about something*’.”

No service user participants reported using the SMR as a method of “up-regulation” to cope with negative symptoms such as fatigue or apathy. However, one health provider participant (occupational therapist) reported using the room as a way to facilitate service user engagement in unit activities, with the ultimate incentive of the service user being able to leave the unit on a pass:

*C8: “So there are a lot of negative symptoms with schizophrenia patients…One of the keys for clients to get on individual or group passes is they need to be engaged on the unit, so [encouraging them to use the sensory room] is one way of me kind of getting them out of their room and using some type of service that we offer*.”

In this example, the sensory room was used to support negative symptoms and up-regulation, as opposed to a method of down-regulation. Yet for most other participants, the value of the room was primarily attributed to supporting down-regulation to help service users deal with feelings such as anger, frustration, anxiety or/and mood fluctuations.

### (3) Alternative to current practices

The third theme was the use of the SMR as an alternative to physical and chemical restraints and/or seclusion. Service users described an intense environment of healing and how it can often be difficult to gather their thoughts and focus on wellness and health. Most service user participants identified that the SMR added value to the care experience by offering another mechanism to manage high levels of anxiety or stress on the unit. One service user reported:

*P3: “The first time I tried the sensory room was I was feeling a bit upset, so I asked for [lorazepam], which is part of the protocol here. So the nurse I talked with said: ‘try the sensory room and see if you still feel emotional about what your thought patterns are’. So I did that and I was in there for a good fifteen minutes, and I came out much calmer, and decided not to pursue the sort of ‘as needed’ medication*.”

Another service user stated:

*P8: “I think that [sensory rooms] should [be used] more in more hospitals instead of giving all these PRNs first.‘Cause they usually give people these PRNs first—like they gave me six Loxapine in one hour and that’s pretty excessive. They could have said go to the sensory room—go lie down [and] relax …but they didn’t do that*”

When another service user participant (P1) was asked if the room had helped them during the stay at the hospital, they responded that the SMR helped by keeping them out of the ‘quiet room’ [i.e. seclusion]. Further, this participant noted that the room provided an outlet for coping and thus reduced their reliance on physical and/or chemical restraints. They also reported a dramatic change in their experiences on the unit simply knowing that the SMR was available.

Some experienced health provider participants reported that service users were learning to advocate for the use of the room itself–specifically to present the argument to staff that the SMR can be an alternative to medications when appropriate. As noted by a health provider:

*C8: “I just see clients going [into the room], being more elevated than we normally see them—if they haven’t had their PRNs or anything like that—and then coming out and easily being engaged with staff. Able to talk to somebody without having pressured speech. Being able to have a conversation with myself or the rehab staff during an arts group or a games group that we have going on—where they’re able to participate because they spent half an hour in the room using the [sensory modalities]*.”

This quote highlights the valued added by the SMR from the perspective of this health provider, as its use optimizes the potential for meaningful and calm patient-provider interactions. In general, both service user and health provider participants viewed the room as a valuable alternative to chemical sedation and/or physical restraints and most participants described the room as a non-invasive, non-pharmacological adjunct to the standard of care.

### (4) Health provider and service user education

The fourth theme was specific to the different participants sampled in this study (i.e. service users and health providers). Within the service user sample, the need for more education about the SMRs and their therapeutic potential was identified. The majority of service user participants (n = 8) reported that they were not introduced to the space by a trained staff member. These participants reported that they knew of the location of the space, its basic intended purpose, and primarily used what was immediately available in the room (e.g. not the modalities located inside the locked cabinet). Service users consistently reported that they would like more training to understand how to maximize the full benefits of the SMR to support their recovery.

*P3*: *“I don’t know what exactly is in that locked cabinet”*, or

*P2*: “*I didn’t know there was stuff in the cupboard*.”

Service users also highlighted how they would like more information regarding how to use the SMR consistently and therapeutically and not only when in crisis:

*P3: “So after that I just now use it automatically if I just need a calming down space …I don’t have to be upset or worried about anything. Initially when I first started using the room a few days ago, I was worried about something. Now I just go there because it’s a nice space …it creates the kind of atmosphere that I want to translate … at home*.”

From the perspective of health providers, a lack of education about the SMR and its therapeutic potential was highlighted. In general, nursing staff reported being educated on the existence of the room via orientation to the unit. However, some nursing staff reported that this orientation has not been comprehensive enough to understand the full potential of the room and the value it added to the patient care experience. One nurse reported:

*C2: “[The room] was part of the tour and I saw others just, kind of, referring to it and stuff, but I think…I don’t know …I think if I had some tangible [information] …looking at the outcomes of [the room] in a formal research way and that you could …actually even discuss with patients too and be like, “hey! studies show that this maybe can be as effective as taking benzo or whatever*.”

One common sub-theme from clinicians was the sentiment that the full potential of the SMR was yet to be maximized. Health providers reported that if they knew more about the SMR, they would be more likely to recommend it patients. One occupational therapist stated:

*C9: “I think there still may be a bit of a lack of education or ongoing education around what the room is used for and stuff in the room, and how we can kind of really use it for each individual patient*.”

Occupational therapists in the study also highlighted a need to learn more about the potential of the room and learn strategies to help service users develop, recover, or maintain participation in meaningful activities and occupations. Another clinician (OT) noted that the training would have to consider the high turnover of staff and the variety of shift-work schedules amongst the different clinical disciplines.

*C6: “So I think [one reason why the staff didn’t find the room useful] can be a lack of education and understanding about what the techniques can be, like how they can help the patient or help anybody, really…. even after a couple of months, you’d have new [group] of casual nurses or …it kind of falls off the radar a little bit …it’s sort of that need for ongoing education*.”

Overall, across service users and health providers, an emergent theme was the need for embedding the clinical utility of the SMR and the evidence supporting it within a program of ongoing education. Service users and nursing staff reported a desire to learn about how to fully engage with the SMR. More specifically, service users reported wanting more knowledge about the specific sensory modalities in the room while nursing staff reported wanting more education with respect to the evidence for the benefits of sensory modulation. Occupational therapists reported a desire for more training the utilization of SMRs to support transition planning and improve outcomes after discharge. Occupational therapists also acknowledged a need for continuing competency training to describe the full rehabilitation value of the SMR in support of the service users’ recovery and re-engagement with meaningful roles and occupations.

## Discussion

Our results suggest overall positive experiences with SMRs in acute tertiary inpatient psychiatry units to enhance care and the service user experience. From the perspective of both service users and health providers, four themes emerged: (1) service users’ empowerment through self-management, (2) emotional regulation, (3) alternative to current practices, and (4) health provider and service user education. These themes were consistent with other literature evaluating the experience of health providers[10] and service users[3, 11]. SMRs were described collectively as a place to learn emotional self-management skills, gain a sense of control, and focus on personal recovery. The acute inpatient setting was identified by most participants as an important platform through which to support the delivery of sensory modulation practices, with emphasis on providing opportunities to learn how to transfer sensory modulation skills into treatment and goal planning for discharge. Yet, from both the service user and health provider perspectives, it was identified that more knowledge was needed to support training and implementation of sensory modulation practices to support their full therapeutic potential.

In this study, SMRs were perceived by both health providers and service users as a safe place for regulating emotions in a busy setting that traditionally has been criticized for its limited capacity to be strength focused and recovery-oriented [36–38]. Although this data emphasized the value of SMRs for down-regulating emotions primarily, understanding and using the full therapeutic potential of SMRs was highlighted by almost all participants (both for personal use and implementation as an evidence-based intervention). All participants identified the need for knowledge about the effectiveness of modalities set up in the SMRs and to have this knowledge effectively transferred to end- users (both service users and health providers). The translation of SMR evidence into practice has also been highlighted by others [14, 39], with emphasis on the need to carefully consider the dynamic nature of the clinical setting in which the SMR is to implemented. Based on these results, early coordinated educational and organizational efforts are needed to optimize the value added by this evidence-based intervention in this setting.

Unique to this study was an emphasis by health providers to systematically use the SMRs to promote transferrable skills post-discharge. Embedding SMRs as part of the care experience was identified as an opportunity to be innovative and strengths-focused in care planning. Although many studies exist to suggest SMRs work at one point in the care journey [11, 12, 14, 40], future research is needed to describe how SMRs can guide recovery-oriented treatment planning throughout the care trajectory, including post-discharge.

Our findings come at an opportune time in regards to the development and implementation of healthcare initiatives in the province of British Columbia [20, 22, 41–43]. With specific reference to mental health care and standards, the Ministry of Health released the Secure Rooms and Seclusion Standards Guidelines in 2012 in an effort to prevent or reduce the use of seclusion rooms and physical restraints[44]. Additionally in 2015, the Mental Health Commission of Canada published the Guidelines for Recovery-Oriented Practice in Canada to operationalize the nature of recovery—both in policy and practice [45–47]. Our findings illustrate that sensory modulation interventions embody principles from these initiatives. Specifically, our data show that service users and health providers view SMRs as an alternative to current practices (including chemical, physical, and environmental restraints), that SMRs promote empowerment through self-management, and instill a sense of hope—all of which are key components of a recovery-oriented framework. This, combined with the findings of similar studies noted above, demonstrates the ecological utility of SMRs in contributing to a recovery-oriented practice. To further highlight this unique point in time, the acute care hospital where this study was conducted is set to be moved and re-constructed, with land procurement and construction to begin this year [48]. Our findings suggest potential efficacy in using sensory-based treatment with patients in psychiatric inpatient care settings. Further research is required to substantiate these findings and the efficacy of SMRs in other acute inpatient psychiatric hospital settings to further understand the diverse experiences of health providers and service users, and to measure the impact of SMRs on quality of care, long-term health outcomes, service utilization and recovery. In addition, there might also be consideration for SMRs in other parts of the hospital (e.g. internal medicine, emergency departments, and rehabilitation medicine) given the high rates of comorbid mental health and substance use disorders.

### Limitations

Our study is not without limitations. The study sample did not constitute a random sample. Health providers were nurses and occupational therapists and did not span the full range of service providers from the units. Service user participants were identified by Clinician Nurse Leaders at one point in time, resulting in a potential sampling bias. Furthermore, the recruitment centre serves a largely inner-city population with high prevalence of homelessness and substance dependence. These factors may limit the generalizability of our findings and highlight the need for further research with a diverse population to validate these results.

As well, despite having collected data on ethnicity, we are did not have a sample size that was large enough to ethically prevent revealing the identification of the participants. Our experience in this study highlighted that SMRs, when incorporated in daily care, may benefit from being aligned with an organization’s cultural safety framework, notably ensuring the needs and priorities of Indigenous people and new comers are met. Future patient-engagement, with people of diverse cultural and ethnic backgrounds is recommended to ensure the SMRs are fit for purpose to support the recovery of any person who chooses to access this service and to understand whether there are socio cultural factors that predict positive outcomes of SMR access and use. Finally, additional work is also needed to better understand how health providers and service users conceptualize the outcomes associated with SMRs, so that the effectiveness and fidelity of SMRs can be explored and compared.
